# Utilizing electronic health record pre-consultation data to create a predictive algorithm for diagnosis of chronic pediatric rheumatic conditions

**DOI:** 10.1007/s10067-025-07631-5

**Published:** 2025-08-16

**Authors:** Kendra R. Lauer, Kyla Driest, Laura R. Pratt, Alysha Taxter

**Affiliations:** 1 Division of Pediatric Rheumatology, Nationwide Children’s Hospital, 405 Butterfly Gardens Drive, Columbus, OH 43215 USA; 2 Department of Pediatrics, The Ohio State University, Columbus, USA; 3 Division of Clinical Informatics, Nationwide Children’s Hospital, 405 Butterfly Gardens Drive, Columbus, OH 43215 USA

**Keywords:** Electronic health record, Juvenile idiopathic arthritis, Pediatric rheumatology, Systemic lupus erythematosus

## Abstract

**Objectives:**

To develop a predictive algorithm for diagnosing chronic pediatric rheumatic conditions using patient-reported, historical, and referral data in the electronic health record (EHR) to address current lengthy consultation wait times.

**Methods:**

All new rheumatology patient evaluations from 2021 to 2023 were retrospectively reviewed to identify the reason for the visit, patient-recorded outcomes, and international classification of disease codes. The data sample was randomly split into 80% derivation and 20% validation sets. Logistic regression evaluated the association of diagnosis and referral data; variables with *p* < 0.2 in univariate were included in a multivariate model. Complete data are reported.

**Results:**

Of the 3139 subjects, 2064 (66%) were female, with a median age of 13 [IQR 8, 16]. Patients diagnosed with inflammatory arthritis numbered 319 (10%), while 55 (2%) were diagnosed with systemic lupus erythematosus (SLE). The median time from the first visit to diagnosing inflammatory arthritis and SLE was 88 days [35, 210] and 42 [17, 132], respectively. In univariate analysis, a referral reason for swelling was positively associated with a new inflammatory arthritis diagnosis. In contrast, antinuclear antibody positivity, rash, and lupus were positively associated with a new SLE diagnosis. Referral data had low sensitivity and high specificity for both inflammatory arthritis and SLE diagnoses, with areas under the curve of 0.59 and 0.65, respectively.

**Conclusion:**

Utilizing the EHR to create a predictive algorithm for chronic rheumatic disease presents a promising solution to existing patient care challenges. This approach suggests that integrating such models to the referral process could help expedite access to pediatric rheumatology services.

**Table:** 

**Key Points** • *Patient referrals to pediatric rheumatology specialists often lead to non-rheumatic diagnosis.* • *Patient-reported data within the electronic health record can be utilized to predict likelihood of rheumatic disease.* • *Electronic algorithms to predict rheumatic disease could expedite patient care access to pediatric rheumatology, which currently has a physician shortage and potentially long wait times.*

**Supplementary Information:**

The online version contains supplementary material available at 10.1007/s10067-025-07631-5.

## Introduction

Compared to other chronic childhood illnesses, pediatric rheumatic disease is relatively rare, affecting an estimated 300,000 children in the United States of America (USA) [1]. Juvenile idiopathic arthritis (JIA) is the most common rheumatic disease of childhood, with an annual incidence of about four per 100,000 children, while systemic lupus erythematosus (SLE) has a yearly incidence of 0.4 per 100,000 [2]. Due to disease rarity, symptoms concerning for an evolving autoimmune disease in a child can be challenging to discern from non-rheumatic etiologies, as symptoms can be non-specific or seemingly unrelated. This non-specificity leads to an unnecessarily high referral rate to pediatric rheumatologists for general symptom complaints that ultimately result in a non-rheumatic diagnosis [2–6]. New rheumatology patients often wait 2–3 months to see a pediatric rheumatologist, and 77% of these referrals will not lead to a diagnosis of a chronic rheumatic condition [5, 7]. Long wait times for initial consultations can put children with genuine rheumatic conditions at risk of developing complications due to untreated disease. These complications may include vision loss from anterior uveitis, growth disturbances and bone erosions for JIA, and chronic kidney disease for SLE [8–10].

Thus, the demand for pediatric rheumatology specialists in the United States is high. As of 2021, the workforce comprised approximately 460 board-certified providers [11]. Compared to other pediatric subspecialties, pediatric rheumatology has one of the smallest workforces per capita of children, with a clinical workforce equivalent of 0.27 providers per 100,000 children nationwide [11, 12]. Geographical location and a relative imbalance between the influx of new graduates and the outflux of retiring providers further impact shortages [13]. Although the workforce is projected to improve to a clinical workforce equivalent of 0.52 by 2040, the number of available providers still remains too low to provide adequate care to children with rheumatic disease [12]. This workforce shortage further contributes to long wait times for initial consultation with providers overwhelmed with referrals for non-specific complaints that infrequently result in a rheumatic diagnosis.

Pre-consultation data would be valuable for performing an initial patient screening to reduce wait times between placing a referral and consultation with a pediatric rheumatologist. A review of referral reasons and chief complaints has previously explored chronic arthritis or systemic inflammatory disease diagnoses [5, 14], but further use of patient-reported data within the electronic health record (EHR) may help predict rheumatic disease before the consultation. Such data may expedite consultation for those whose assessments are most concerning for new rheumatic disease, negate the necessity of some referrals, and alleviate the current workforce demand for pediatric rheumatologists. This study aims to develop a predictive algorithm for diagnosing chronic inflammatory arthritis and SLE using patient-reported, historical, and referral data available within the EHR pre-consultation.

## Materials and methods

### Study population

New patients evaluated between 2021 and 2023 in the outpatient rheumatology clinic at Nationwide Children’s Hospital were included. Visit types of “New Patient” (utilized when they have established care with an outside pediatric rheumatologist) and “Consultation” (utilized when a patient has not established care with rheumatologist) define a new patient and are guided by scheduling templates and programming rules; patients which have been previously seen within the past 3 years by a pediatric rheumatologist at our institution are scheduled using “Return Visit” type.

### Patient characteristics

Age and sex at birth were extracted from the EHR.

### Visit characteristics

The administrative staff record “Reason for referral” during the scheduling process; reasons were categorized into positive antinuclear antibodies (ANA), pain or stiffness (i.e., pain, arthropathy, arthralgia, myalgia, limp, stiff, fibromyalgia), swelling (i.e., arthritis, sacroiliitis, effusion, synovitis, juvenile idiopathic arthritis (JIA)), fever (i.e., febrile), dermatologic concern (i.e., skin, alopecia, urticaria, livedo, dermatitis, petechiae, hives), and lupus (i.e., SLE). Manually reviewing the reasons accounted for misspellings.

The nursing team enters a “Reason for the visit” during rooming. Reasons were categorized into consult/new patient, pain complaint (i.e., ankle pain, back pain, muscle pain, musculoskeletal pain, abdominal pain, fibromyalgia), laboratory problem (i.e., abnormal lab results, elevated lab test, lab follow up, results), arthritis (i.e., arthritis, JIA, swelling), joint or musculoskeletal problems (i.e., finger problem, gait abnormality, enthesitis, Ehlers Danlos Syndrome), dermatologic/skin problem (i.e., rash, hives, bruising, psoriasis, scleroderma, skin problem), eye concerns (i.e., eye problem, uveitis), fatigue, oral and ear, nose, throat concerns (i.e., hearing loss, mouth problem, nosebleed, oral lesion, ulcer), fevers (i.e., fever, recurrent fevers), gastrointestinal (i.e., diarrhea, inflammatory bowel disease), neurologic concern (sleep problem, headaches), cardiac concern (i.e., aortic dilation, dizziness, postural orthostatic tachycardia syndrome, swelling, syncope, vasculitis), other (i.e., autoimmune disease, follow up, joint injection, lupus, other problem, teaching), or gynecologic (i.e., heavy menses). Patients could have more than one recorded reason for the visit.

### Patient-reported outcomes

All patients complete electronic questionnaires during routine clinical care. An electronic review-of-systems (ROS) questionnaire, 55-point (for boys) or 56-point (for girls), surveying the domains of constitutional, eye, ear, nose, mouth, throat, cardiovascular, respiratory, gastrointestinal, urinary, reproductive, muscular, joint, skin, neurologic, and psychologic symptoms was provided. Physical function was measured using the Childhood Health Assessment Questionnaire, with a higher score indicating more impaired physical function. Pain was rated 0–10, with a higher score indicating more pain. Patient global disease assessment was rated 0–10, with a higher score indicating worse disease. The presence or absence of morning stiffness was assessed. The ability to manage the condition was rated 0–10, with a higher score indicating more confidence. When patients did not or could not complete questionnaires, the provider captured these data and entered them into the EHR.

### Provider-reported outcomes

All joints were examined during routine clinical care and were documented on a standardized template. The number of painful, swollen, and active joints (defined as either swollen or painful with decreased motion) was recorded. Provider global disease assessment was rated 0–10, with higher scores indicating worse disease.

### JIA disease activity score

The clinical juvenile arthritis disease activity score (cJADAS) is a composite score for children diagnosed with arthritis that is calculated by the sum of the patient global disease assessment score, provider global disease assessment score, and active joint count (with a maximum of 10 joints) [15, 16].

### Diagnosis of rheumatic condition

Initial and future encounter diagnoses within pediatric rheumatology clinic, medical history, and problem list International Classification of Disease 10 (ICD-10) codes were categorized into diagnosis of chronic inflammatory arthritis [L40.5 (psoriatic arthritis), M05 (rheumatoid arthritis), M06 (inflammatory polyarthropathy), M08 (JIA), M45 (ankylosing spondylitis (AS))] and lupus [M32 (lupus)]. The diagnosis date was recorded. A diagnosis of chronic inflammatory arthritis or lupus was assumed if a patient had one or more diagnosis codes documented during a pediatric rheumatology visit.

### Statistical analysis

Summary statistics, including count (percent) and median [interquartile range (IQR)], described the patient cohort. Chi-square or Fisher exact evaluated the association between the reason for referral and diagnosis for small samples. The Wilcoxon signed-rank test assessed the association between continuous variables and diagnosis outcome. Statistical significance is defined as *p* < 0.05.

### Model development

A logistic regression evaluated the association of a rheumatic diagnosis with demographics, referral reason, and patient-reported data. The sample was randomly split into 80% as a training set and 20% as a validation set. The referral reason and review of system variables with *p* < 0.2 in univariate analysis were included in multivariate models. A priori, variables not clinically thought to be associated with an outcome, including urinary symptoms for a new JIA diagnosis, were excluded from multivariable models. The final multivariate model was evaluated on the validation set. Odds ratio (OR) and 95% confidence intervals (CI) are reported.

### Model performance

Multiple cutoffs for our validation logistic regression models were evaluated; the positive outcome probability threshold was set to 0.05 to maximize specificity and to exclude those without a rheumatic condition, as all patients would eventually receive a rheumatology appointment, and our goal was to identify who would benefit from an expedited appointment given higher concern for a true chronic rheumatic condition. Sensitivity, specificity, positive predictive value (PPV), negative predictive value (NPV), and area under the curve (AUC) set models are reported.

### Sensitivity analysis

Sensitivity analysis was conducted by including only those patients with chronic inflammatory arthritis or lupus who had at least four encounter diagnoses for the respective condition within the pediatric rheumatology clinic [17, 18]. The positive outcome probability threshold for sensitivity analysis was set to 0.20.

## Results

The study period included 3139 new rheumatology patient visits; 2064 (66%) were female, and the median age was 13. The most common reasons for referral were pain (39%), swelling (15%), and ANA positive (11%) (Table 1). Nursing documented reason for visit was “Consult” for 3018 (96%) of visits; all other individual reasons for visit categories were documented in < 1% of visits.

**Table 1:** Demographics and reason for referral by future diagnosis

	All patients *N* = 3139	New chronic inflammatory arthritis diagnosis *N* = 148	New SLE diagnosis *N* = 19
Age, years, median [SD]	13	13	15^a^
Female sex	2064 (66%)	93 (63%)	15 (79%)
**Referral reason:**			
Pain	1232 (39%)	62 (41%)	4 (21%)^a^
Swelling	479 (15%)	58 (39%)^a^	2 (10%)
ANA positive	336 (11%)	9 (6%)	5 (26%)^a^
Rash	227 (7%)	7 (5%)	4 (21%)^a^
Fever	211 (7%)	3 (2%)^a^	0 (0%)
SLE	74 (2%)	3 (2%)	3 (15%)^a^

Fisher exact used for SLE comparisons

*SD* standard deviation, *ANA* antinuclear antibody, *SLE* systemic lupus erythematosus

^a^*p* < 0.05

### Diagnosis of chronic inflammatory arthritis

Children diagnosed with chronic inflammatory arthritis during the study period totaled 319 (10%), of which 171 (5%) were diagnosed before establishing rheumatology care. The median time from the first rheumatology visit to establishing a new diagnosis of chronic inflammatory arthritis was 88 days [35, 210]. The median provider global disease assessment score was 3, and the median cJADAS score was 9. Children with a new diagnosis of chronic inflammatory arthritis were more frequently referred for swelling and less frequently for fever. The physical function scores were worse, the pain higher, and the patient’s global score was higher. Morning stiffness was more likely to be reported for new chronic inflammatory arthritis diagnoses than those without a new diagnosis. Children with a new chronic inflammatory arthritis diagnosis reported cardiac or neurologic symptoms less commonly and reported joint symptoms more commonly (Table 2).

**Table 2:** Patient-reported outcomes by future diagnosis

	All patients *N* = 3139	New chronic inflammatory arthritis diagnosis *N* = 148	New SLE diagnosis *N* = 19
Physical function^a^	0.25 [0, 0.875]	0.625 [0.25, 1.125]^b^	0.5 [0.25, 1.125]
Pain^c^	4	6^b^	7^b^
Patient global^d^	4	5^b^	4
Morning stiffness^e^	1260 (62%)	77 (82%)^b^	10 (83%)
Ability to manage condition^f^	10	10^b^	10
**ROS domain**			
Constitutional	1470 (47%)	65 (44%)	11 (58%)
Eye	732 (23%)	38 (26%)	6 (32%)
Ear	432 (14%)	17 (12%)	2 (11%)
Nose	368 (12%)	18 (12%)	3 (16%)
Mouth	889 (28%)	44 (27%)	5 (27%)
Cardiac	815 (26%)	24 (16%)^b^	7 (37%)
Respiratory	614 (20%)	31 (21%)	4 (21%)
Gastrointestinal	1059 (34%)	46 (31%)	6 (32%)
Urinary	257 (8%)	16 (11%)	3 (16%)
Reproductive	246 (8%)	8 (5%)	3 (16%)
Joint	1459 (46%)	83 (56%)^b^	10 (53%)
Muscle	1158 (37%)	49 (33%)	9 (47%)
Skin	837 (27%)	32 (22%)	11 (58%)^b^
Hematologic	643 (20%)	29 (20%)	5 (26%)
Neurologic	1069 (34%)	35 (24%)^b^	6 (32%)
Psychologic	1187 (38%)	48 (32%)	9 (47%)
Total positive ROS	7	6	11^b^

*ROS* review of systems, *SLE* systemic lupus erythematosus

^a^Available in 2151 subjects

^b^*p* < 0.05

^c^Available in 2059 subjects

^d^Available in 2176 subjects

^e^Available in 2024 subjects

^f^Available in 1760 subjects

### Predictive modeling for chronic inflammatory arthritis

**Fig. 1:**
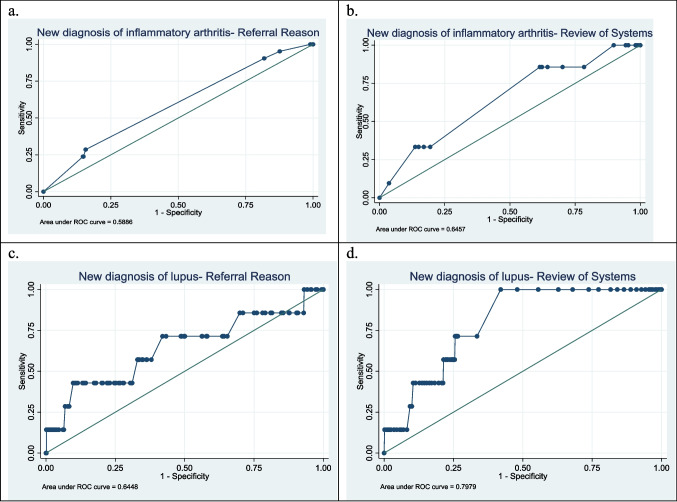
Receiver operator curves (ROC) evaluates multivariate models of **a** referral reasons of antinuclear antibody positivity, swelling, and fever with a new diagnosis of chronic inflammatory arthritis. **b** Positive cardiology, joint, dermatologic, and neurologic review-of-systems (ROS) concern with a new diagnosis of chronic inflammatory arthritis. **c** Referral reasons of antinuclear antibody positivity, rash, and lupus concerns, age, and sex with a new diagnosis of systemic lupus erythematosus. **d** Total positive ROS count and positive dermatologic ROS concern with a new diagnosis of systemic lupus erythematosus

In univariate analysis, referral reasons of swelling was associated with a new diagnosis of chronic inflammatory arthritis (OR 4.38 (3.01, 6.35), *p* < 0.01) (Table 3). Fever was negatively associated with a new diagnosis of chronic inflammatory arthritis (OR 0.21, (0.05, 0.87) *p* = 0.03). A review of system domains of cardiac and neurologic symptoms was protective against a new diagnosis of chronic inflammatory arthritis, while joint symptoms were associated with a new diagnosis of chronic inflammatory arthritis. None of the referral reason variables was statistically significant in multivariate analysis within the validation set (Table 4); a model evaluating the association of chronic inflammatory arthritis and referral reasons had a sensitivity of 24%, specificity of 85%, and AUC of 0.59 (Fig. 1a). A multivariable model evaluating the association of chronic inflammatory arthritis and ROS showed neurologic symptoms were protective against a diagnosis of chronic inflammatory arthritis (Table 5); a model showed 33% sensitivity, 85% specificity, and an AUC of 0.65 (Fig. 1b).

**Table 3:** Factors associated with a new diagnosis of rheumatic condition: training set

	New chronic inflammatory arthritis diagnosis odds ratio (95% CI)	*p*-value	New SLE diagnosis odds ratio (95% CI)	*p*-value
Age	0.99 (0.96, 1.03)	0.80	1.11 (1.02, 1.21)	0.01
Female sex	1.07 (0.73, 1.54)	0.74	0.17 (0.02, 1.31)	0.09
**Referral reason**				
ANA positive	0.50 (0.23, 1.09)	0.08	4.50 (1.34, 15.04)	0.02
Pain	0.95 (0.66, 1.37)	0.79	0.51 (0.14, 1.87)	0.31
Swelling	4.38 (3.01, 6.35)	< 0.01	0.51 (0.07, 3.98)	0.52
Fever	0.21 (0.05, 0.87)	0.03	–	–
Rash	0.64 (0.27, 1.48)	0.30	4.45 (1.19, 16.60)	0.03
SLE	1.00 (0.31, 3.25)	0.99	8.56 (1.83, 39.99)	< 0.01
**Patient reported outcomes**				
Physical function	1.88 (1.38, 2.55)	< 0.01	1.53 (0.56, 4.20)	0.41
Pain	1.15 (1.06, 1.24)	< 0.01	1.57 (1.10, 2.21)	0.01
Patient global	1.18 (1.09, 1.28)	< 0.01	1.20 (0.93, 1.55)	0.17
Morning stiffness	2.52 (1.46, 4.33)	< 0.01	–	–
Ability to manage condition	1.14 (0.97, 1.33)	0.10	0.87 (0.69, 1.10)	0.24
Total positive ROS	1.00 (0.98, 1.02)	0.56	1.03 (0.98, 1.08)	0.20
**ROS domain**				
Constitutional	0.89 (0.62, 1.27)	0.52	1.59 (0.50, 5.03)	0.43
Eye	1.08 (0.72, 1.63)	0.72	1.06 (0.29, 3.94)	0.93
Ear	0.88 (0.51, 1.50)	0.64	1.23 (0.27, 5.63)	0.79
Nose	1.03 (0.60, 1.76)	0.92	0.65 (0.08, 5.02)	0.68
Mouth	1.08 (0.73, 1.59)	0.70	0.50 (0.11, 2.29)	0.37
Cardiac	0.53 (0.33, 0.86)	0.01	1.39 (0.42, 4.62)	0.60
Respiratory	1.01 (0.65, 1.59)	0.95	1.38 (0.37, 5.12)	0.62
Gastrointestinal	0.92 (0.63, 1.35)	0.68	0.65 (0.17, 2.39)	0.51
Urinary	1.64 (0.95, 2.82)	0.07	2.21 (0.48, 10.17)	0.31
Reproductive	0.76 (0.37, 1.58)	0.46	2.30 (0.50, 10.57)	0.29
Joint	1.60 (1.11, 2.30)	0.01	1.16 (0.37, 3.60)	0.80
Muscle	0.93 (0.64, 1.35)	0.71	1.71 (0.55, 5.31)	0.36
Skin	0.74 (0.48, 1.13)	0.17	2.78 (0.89, 8.65)	0.08
Hematologic	1.04 (0.67, 1.60)	0.88	1.28 (0.34, 4.74)	0.71
Neurologic	0.64 (0.43, 0.97)	0.04	0.65 (0.18, 2.40)	0.52
Psychologic	0.90 (0.62, 1.31)	0.59	1.18 (0.37, 3.73)	0.78

*ANA* antinuclear antibody, *SLE* systemic lupus erythematosus, *ROS* review of systems

**Table 4:** Odds of referral reason with new diagnosis of rheumatic condition: test set

	New chronic inflammatory arthritis diagnosis Odds ratio (95% CI)	*p*-value	New SLE diagnosis^a^	*p*-value
**Referral reason**				
ANA positive	0.76 (0.17, 3.37)	0.72	0.87 (0.09, 8.05)	0.91
Pain	–	–	–	–
Swelling	2.07 (0.78, 5.56)	0.15	–	–
Fever	0.78 (0.10, 6.02)	0.81	–	–
Rash	–	–	1.98 (0.22, 17.51)	0.54
SLE	–	–	8.41 (0.86, 91.97)	0.07
Sensitivity	24%		15%	
Specificity	85%		98%	
PPV	5%		8%	
NPV	97%		99%	
Correctly classified	83%		97%	
AUC	0.59		0.64	

*ANA* antinuclear antibody, *SLE* systemic lupus erythematosus, *PPV* positive predictive value, *NPV* negative predictive value, *AUC* area under the curve

^a^Adjusted for age and sex

**Table 5:** Odds of positive review of systems with new diagnosis of rheumatic condition: test set

	New chronic inflammatory arthritis diagnosis Odds ratio (95% CI)	*p*-value	New SLE diagnosis odds ratio (95% CI)	*p*-value
**ROS domain**				
Total positive ROS			0.98 (0.90, 1.07)	0.73
Cardiac	0.75 (0.19, 2.99)	0.68		
Joint	1.86 (0.69, 5.00)	0.22		
Skin	1.25 (0.37, 4.00)	0.71	8.22 (1.23, 54.75)	0.03
Neurologic	0.22 (0.05, 0.90)	0.04		
Sensitivity	33%		0%	
Specificity	85%		100%	
PPV	7%		–	
NPV	97%		99%	
Correctly classified	83%		99%	
AUC	0.65		0.80	

*ROS* review of systems, *SLE* systemic lupus erythematosus, *PPV* positive predictive value, *NPV* negative predictive value, *AUC* area under the curve

### Diagnosis of SLE

Children diagnosed with SLE numbered 55 (2%) during the study period, including 36 (1%) diagnosed before establishing care. The median time from the first visit to establishing a new diagnosis of SLE was 42 days [17, 132]. The median provider global disease assessment score was 1 [0, 5]. Patients newly diagnosed with SLE frequently had referral reasons for positive ANA, rash, or lupus, and were less commonly referred for pain (Table 1). Both patient-reported pain and positive total review-of-systems domains in children with a new SLE diagnosis were higher. Children with a new diagnosis of SLE were more likely to report skin symptoms (Table 2).

### Predictive modeling for SLE

Referral reasons for ANA positivity, rash, and lupus were positively associated with a new diagnosis of SLE in the training set (Table 3). The training set had no statistically significant ROS domains associated with a diagnosis of SLE. None of the referral reason variables was statistically significant in a multivariate analysis within the validation set (Table 4); a model using referral reason had a sensitivity of 15%, specificity of 98%, and AUC of 0.64 (Fig. 1c). A multivariable model evaluating ROS showed that patient-reported skin symptom was associated with a diagnosis of SLE (Table 5), with the final model showing a sensitivity of 0%, specificity of 100%, and AUC of 0.80 (Fig. 1d).

### Sensitivity analysis

For patients with four or more encounter diagnosis of inflammatory arthritis (*N* = 241), the original referral reason variables of ANA positivity, swelling, and fever, as well as additional variables of pain and rash, had *p* < 0.2 in univariate analysis (Supplemental Table 1). Multivariable model evaluating referral reason for swelling was positively associated with a new diagnosis of inflammatory arthritis (OR 3.74 (1.98, 7.08), *p* < 0.01), with the final model showing a sensitivity of 22%, specificity of 92%, and AUC of 0.67.

Similarly, the four initial ROS variables, as well as constitutional, ear, reproductive, and psychologic symptoms had a *p* < 0.2 in univariate analysis (Supplemental Table 2). Multivariable model evaluating ROS domain of joint was associated with a new diagnosis of chronic inflammatory arthritis (OR 2.28 (1.03, 5.07), *p* = 0.04), with a final model showing a sensitivity of 4%, specificity of 98%, and AUC of 0.63.

For patients with four or more encounter diagnosis of SLE (*N* = 27), referral reasons for ANA positivity and lupus, as well as pain, had a *p* < 0.2 in univariate analysis. No variables were significant in multivariable modeling, with a final model showing a sensitivity of 0%, specificity of 100%, and AUC of 0.50. Similarly, patient-reported skin concerns persisted, and no additional variables had a *p* < 0.2 in univariate analysis. Multivariable model evaluating the ROS domain of skin concerns was associated with a new diagnosis of SLE (OR 7.11 (1.28, 39.28), *p* = 0.03), with a final model showing a sensitivity of 0%, specificity of 100%, and AUC of 0.70.

## Discussion

To the best of our knowledge, this paper is one of the first to evaluate how data collected before and during routine clinical care—specifically, information documented in the EHR, including referral reason and patient-entered data—can be used to develop a predictive diagnostic algorithm. This algorithm aims to identify children who may have a chronic rheumatic condition using information available before and at the initial pediatric rheumatology consultation visit. Notably, this data can be collected before a child meets with a provider and can help inform clinical decision-making, potentially even before the child arrives at the clinic. Gathering patient information before consultation may enable us to develop models within the EHR to prioritize referrals, especially during pediatric rheumatologist shortages, and expedite treatment.

Previous studies have evaluated pediatric rheumatology patients’ chief complaints and referral reasons to diagnose rheumatic disease [3, 5, 14], but other patient-reported outcomes have not been used. Patient-reported outcomes collected during routine pediatric rheumatology clinic visits, e.g., the Childhood Health Assessment Questionnaire, ROS, and patient global disease assessment score at our institution, offer valuable insights into patient-experienced symptoms. This information can be instrumental in evaluating rheumatic diseases. Our model found that data commonly available before a visit, such as the reason for referral and patient-entered data, can be highly specific but not sensitive when creating a predictive model for a new diagnosis of chronic inflammatory arthritis and lupus. While screening algorithms often use high-sensitivity protocols to capture all potential cases, our algorithm focuses on high specificity. Minimizing false positives is essential during triage to prevent unnecessary use of limited resources and to ensure timely evaluation of patients most likely to have a chronic rheumatic condition. As such, high specificity is a clinically valuable feature, even when sensitivity is low, as observed in our models, which achieved sensitivities of only 24% and 15% for chronic inflammatory arthritis and lupus, respectively, which typically had minimal changes when evaluated in sensitivity analysis, although limited to small sample size. It identifies symptoms and disease characteristics more indicative of rheumatologic conditions enabling faster patient initial rheumatology consultations. This specificity allows a more targeted screening while avoiding overburdening of every potential positive screen.

Diagnosis of chronic inflammatory arthritis during our study period was seen in approximately 10% of our patients, with half having a pre-existing diagnosis of chronic inflammatory arthritis before the consultation. The most commonly reported symptoms for referral for concern of chronic inflammatory arthritis included joint pain, swelling, and positive ANA. Children diagnosed with chronic inflammatory arthritis were more likely to report joint swelling and morning stiffness as a presenting complaint. These findings were consistent with similar prior studies [3, 14]. Joint pain remains a non-specific symptom with a low association with chronic inflammatory arthritis diagnoses in the absence of joint swelling and stiffness, reiterating that joint pain is more likely to have a negative predictive value for the presence of chronic inflammatory arthritis or rheumatic disease. Providers who do not specialize in bone or joint conditions have lower confidence in their musculoskeletal exam than in their cardiovascular, respiratory, or abdominal exam [19, 20], which may explain why many joint pain referrals result in a non-rheumatic disease diagnosis.

SLE was diagnosed in approximately 2% of patients during our study period, with half already having an established diagnosis before rheumatology consultation. The median time to diagnosis was about half of that seen in the chronic inflammatory arthritis cohort. Pediatric SLE often presents with more severe phenotypes with aggressive disease than their adult counterparts [21–23]. Given this aggressive nature, one can presume that children with new onset pediatric SLE are more likely to present sooner to a primary care provider or require hospital admission due to acute progression of symptoms, resulting in an SLE diagnosis before initial outpatient rheumatology consultation. Referral for a positive ANA was also positively associated with a new diagnosis of SLE in our study, consistent with a prior study showing ANA positivity and high titers having a PPV for SLE versus chronic inflammatory arthritis counterparts [24]. Interestingly, skin symptoms were the only patient-reported variable significant for predicting a new diagnosis of SLE. Acute skin diseases, such as malar rash, generalized lupus rash, cutaneous vasculitis, and photosensitivity, are more commonly observed at the onset of pediatric SLE than in adults. Thus, acute skin symptoms may be a significant indicator when evaluating patient-reported data for suspicion of pediatric SLE [25, 26].

This study reinforces the limited usefulness of a screening ANA test. Our findings indicate that ANA positivity was not linked to a diagnosis of chronic inflammatory arthritis when analyzed using univariate or multivariate modeling. While the ANA test was associated with an SLE diagnosis in univariate analysis, it was not in multivariate modeling. Numerous studies have evaluated the ANA test for adult and pediatric autoimmune diseases. However, ANA results have consistently shown low diagnostic utility and a low PPV when used as both a diagnostic test and screening tool for autoimmune disease in children with musculoskeletal complaints [24, 27–29]. A recent study evaluating adult referrals to rheumatology noted that ANA testing was obtained without clinical indication in 20% of new referrals, which ultimately led to unnecessary referrals, patient anxiety, and increased costs to the patient’s family [30]. The American College of Rheumatology Choosing Wisely campaign has addressed these challenges by recommending that antibody testing should not be ordered without evidence of rheumatic disease [31]. This study supports prior findings regarding the limited utility of the ANA test in diagnosing autoimmune disease and how this marker should not be obtained as a screening tool.

We observed patient consultation wait times to be about 3 months, consistent with similar studies [7, 32–34]. Unfortunately, these prolonged wait times for pediatric rheumatology patient referrals are typical due to our current workforce shortage. This issue has been seen in the United States and worldwide, with wait times as long as 30 months in Saudi Arabia [3]. Provider recognition of autoimmune disease can also impact the time it takes for patients to see a pediatric rheumatologist. Children with chronic musculoskeletal complaints are more commonly referred to primary care physicians, emergency medicine physicians, and orthopedic specialists for consultation due to the higher prevalence of non-rheumatic conditions [34]. Unfortunately, this practice leads to delays in care for those with true chronic inflammatory arthritis, resulting in unnecessary invasive procedures and increased risk of complications due to prolonged untreated disease [35]. Europe has implemented initiatives to expedite rheumatology referrals. The European Alliance of Associations for Rheumatology recommends that a rheumatologist evaluate for inflammatory arthritis within 6 weeks after symptom onset [36]. The British Society for Paediatric and Adolescent Rheumatology in the UK has guidelines for pediatric rheumatology consultation within 10 weeks of symptom onset and 4 weeks of referral [37]. A follow-up study evaluating these guidelines’ effectiveness showed variable clinical use of guidelines, with about 40% of patients seen within 10 weeks of symptom onset and 60% seen within 4 weeks of referral [38]. Additional studies have also demonstrated that starting treatment earlier for inflammatory arthritis improves disease outcomes, highlighting the urgent need to reduce long wait times to enhance clinical outcomes [39, 40].

This study must be interpreted with several limitations. Our study was performed at one academic quaternary medical center with a retrospective review over a 3-year period without external validation. We leveraged real-world data from our EHR. However, diagnoses could have been incorrectly entered, such as a primary care provider entering a chronic arthritis diagnosis, whereas a rheumatologist would recognize these symptoms as arthralgia and not arthritis. Referral reasons for rheumatology consultation may have also been lost during the scheduling or triaging of patients, given the current lack of available scheduling or charting options for both administrative and nursing staff, resulting in the reason being listed as “consultation.” SLE diagnosis at initial outpatient consultation was lower than expected, presumed due to most new-onset pediatric SLE having more acute onset of symptoms, prompting sooner medical evaluation and higher suspicion for SLE by providers compared to adult counterparts [21–23], resulting in a diagnosis before the initial consultation. Our study only evaluated the diagnosis of two pediatric rheumatologic conditions, chronic inflammatory arthritis and SLE, without identifying other rheumatic or non-rheumatic diagnoses within the study population, such as acute arthritis or chronic musculoskeletal pain. While the referral provider’s specialty was not identified, obtaining this data could shed light on current referral patterns to pediatric rheumatology and improve wait times [3, 4, 7, 34]. Additionally, referral reasons were not standardized and were manually entered, taking care to account for misspellings and synonyms.

Using pre-consultation data for predicting chronic rheumatic disease diagnoses in children should be further explored. It would be beneficial to conduct additional prospective studies over a more extended period using a developed algorithm within the EHR for patient triage. Referral triage has been successfully implemented within adult rheumatology, as evidenced by decreased wait times and improved predictive accuracy for new rheumatic disease [32, 41]. Further evaluation could also include other pediatric rheumatic conditions, such as juvenile dermatomyositis, and non-rheumatic conditions, such as biomechanical and musculoskeletal joint pain, fibromyalgia, and amplified pain.

## Conclusion

Utilizing data within the EHR to create a predictive algorithm for diagnosing chronic rheumatic disease prior to pediatric rheumatology consultation is a promising tool to address current barriers to timely patient care access. Pre-consultation, patient-reported data can provide valuable insight into predicting the likelihood of rheumatic disease. The challenges of referring providers to recognize childhood rheumatic diseases early result in unnecessary referrals and lengthy wait times to pediatric rheumatology. The current pediatric rheumatology workforce shortage widens this gap, placing children with rheumatic disease at risk for worse disease outcomes and delays in care. Our data suggest that integrating such models into the referral process could expedite access for children with high suspicion of rheumatic disease to pediatric rheumatology.

## Supplementary Information

Below is the link to the electronic supplementary material.

Supplementary file1 (DOCX 19 KB)

Supplementary file2 (DOCX 19 KB)

## Data Availability

The datasets generated during and/or analyzed during the current study are available from the corresponding author on reasonable request.

## Abbreviations

ANA: Antinuclear antibodies
AUC: Area under the curve
CI: Confidence interval
cJADAS: Clinical juvenile arthritis disease activity score
EHR: Electronic health record
ICD-10: International Classification of Disease 10
IQR: Interquartile range
JIA: Juvenile idiopathic arthritis
NPV: Negative predictive value
OR: Odds ratio
PPV: Positive predictive value
ROS: Review-of-systems
SLE: Systemic lupus erythematosus
