# Supplementary material for: Utilizing electronic health record pre-consultation data to create a predictive algorithm for diagnosis of chronic pediatric rheumatic conditions
Source: Clin Rheumatol. 2025 Aug 16;44(10):4203–14. doi: 10.1007/s10067-025-07631-5 (PMC12518392; doi:10.1007/s10067-025-07631-5)
Supplement: Supplementary file 1 — Supplementary file1 (DOCX 19 KB) [file 10067_2025_7631_MOESM1_ESM.docx]

**Supplemental Table 1: Factors Associated with a New Diagnosis of Rheumatic Condition by Referral Reason: Sensitivity Analysis**

|  | **Training** | | **Testing** | | **Training** | | **Testing** | |
| --- | --- | --- | --- | --- | --- | --- | --- | --- |
|  | **New Chronic Inflammatory Arthritis Diagnosis**  **Odds Ratio (95%CI)** | **p-value** | **New Chronic Inflammatory Arthritis Diagnosis**  **Odds Ratio (95%CI)** | **p-value** | **New SLE Diagnosis Odds Ratio (95%CI)** | **p-value** | **New SLE Diagnosis Odds Ratio (95%CI)** | **p-value** |
| **Referral Reason** |  |  |  |  |  |  |  |  |
| ANA positive | 0.31 (0.14, 0.67) | <0.01 | 0.33 (0.08, 1.43) | 0.14 | 2.11 (0.71, 6.31) | 0.18 | 1.04 (0.12, 9.12) | 0.98 |
| Pain | 0.64 (0.46, 0.87) | <0.01 | 0.87 (0.46, 1.65) | 0.67 | 0.25 (0.07, 0.85) | 0.02 | -- | -- |
| Swelling | 6.04 (4.44, 8.21) | <0.01 | 3.75 (1.98, 7.07) | <0.01 | 0.59 (0.14, 2.55) | 0.48 | -- | -- |
| Fever | 0.13 (0.03, 0.54) | <0.01 | -- | -- | -- | -- | -- | -- |
| Rash | 0.40 (0.17, 0.91) | 0.03 | 0.85 (0.25, 2.92) | 0.80 | 0.66 (0.09, 4.93) | 0.68 | -- | -- |
| SLE | 0.86 (0.30, 2.40) | 0.78 | -- | -- | 23.44 (9.08, 60.49) | <0.01 | -- | -- |
| Sensitivity | -- | -- | 22% | -- | -- | -- | -- | -- |
| Specificity | -- | -- | 92% | -- | -- | 100% | -- | -- |
| PPV | -- | -- | 18% | -- | -- | -- | -- | -- |
| NPV | -- | -- | 93% | -- | -- | 98% | -- | -- |
| Correctly Classified | -- | -- | 86% | -- | -- | 98% | -- | -- |
| AUC | -- | -- | 0.67 | -- | -- | 0.50 | -- | -- |

Legend: **ANA**: antinuclear antibody. **ROS**: Review of systems. **SLE**: systemic lupus erythematosus.
