# Supplementary material for: Utilizing electronic health record pre-consultation data to create a predictive algorithm for diagnosis of chronic pediatric rheumatic conditions
Source: Clin Rheumatol. 2025 Aug 16;44(10):4203–14. doi: 10.1007/s10067-025-07631-5 (PMC12518392; doi:10.1007/s10067-025-07631-5)
Supplement: Supplementary file 2 — Supplementary file2 (DOCX 19 KB) [file 10067_2025_7631_MOESM2_ESM.docx]

**Supplemental Table 2: Factors Associated with a New Diagnosis of Rheumatic Condition by Review of Systems: Sensitivity Analysis**

|  | **Training** | | **Testing** | | **Training** | | **Testing** | |
| --- | --- | --- | --- | --- | --- | --- | --- | --- |
|  | **New Chronic Inflammatory Arthritis Diagnosis**  **Odds Ratio (95%CI)** | **p-value** | **New Chronic Inflammatory Arthritis Diagnosis**  **Odds Ratio (95%CI)** | **p-value** | **New SLE Diagnosis Odds Ratio (95%CI)** | **p-value** | **New SLE Diagnosis Odds Ratio (95%CI)** | **p-value** |
| **ROS Domain** |  |  |  |  |  |  |  |  |
| Constitutional | 0.72 (0.54, 0.97) | 0.03 | 1.16 (0.50, 2.68) | 0.72 | 1.03 (0.44, 2.44) | 0.94 | -- | -- |
| Eye | 0.93 (0.65, 1.31) | 0.66 | -- | -- | 0.75 (0.25, 2.24) | 0.61 | -- | -- |
| Ear | 0.72 (0.45, 1.15) | 0.18 | 0.59 (0.19, 1.83) | 0.37 | 1.93 (0.70, 5.30) | 0.20 | -- | -- |
| Nose | 0.75 (0.46, 1.22) | 0.26 | -- | -- | 1.19 (0.35, 4.06) | 0.78 | -- | -- |
| Mouth | 0.93 (0.67, 1.29) | 0.69 | -- | -- | 0.78 (0.29, 2.15) | 0.63 | -- | -- |
| Cardiac | 0.46 (0.30, 0.69) | <0.01 | 0.70 (0.27, 1.81) | 0.46 | 0.86 (0.32, 2.37) | 0.77 | -- | -- |
| Respiratory | 1.07 (0.74, 1.54) | 0.70 | -- | -- | 0.97 (0.33, 2.90) | 0.96 | -- | -- |
| GI | 0.85 (0.63, 1.17) | 0.32 | -- | -- | 0.77 (0.30, 2.00) | 0.60 | -- | -- |
| Urinary | 0.84 (0.47, 1.18) | 0.55 | -- | -- | 1.85 (0.54, 6.33) | 0.33 | -- | -- |
| Reproductive | 0.53 (0.27, 1.05) | 0.07 | 1.27 (0.33, 4.88) | 0.73 | 0.57 (0.07, 4.25) | 0.58 | -- | -- |
| Joint | 1.44 (1.07, 1.94) | 0.01 | 2.28 (1.03, 5.07) | 0.04 | 1.55 (0.65, 3.68) | 0.32 | -- | -- |
| Muscle | 0.90 (0.66, 1.22) | 0.52 | -- | -- | 1.28 (0.54, 3.05) | 0.58 | -- | -- |
| Skin | 0.71 (0.50, 1.02) | 0.07 | 1.56 (0.71, 3.48) | 0.27 | 2.08 (0.88, 4.98) | 0.10 | 7.11 (1.28, 39.28) | 0.03 |
| Hematologic | 0.95 (0.66, 1.37) | 0.80 | -- | -- | 1.19 (0.44, 3.29) | 0.73 | -- | -- |
| Neurologic | 0.50 (0.35, 0.70) | <0.01 | 0.58 (0.24, 1.41) | 0.23 | 0.60 (0.22, 1.67) | 0.33 | -- | -- |
| Psychologic | 0.81 (0.60, 1.11) | 0.19 | 0.45 (0.19, 1.06) | 0.07 | 1.24 (0.52, 2.96) | 0.62 | -- | -- |
| Sensitivity | -- | -- | 4% | -- | -- | -- | 0% | -- |
| Specificity | -- | -- | 98% | -- | -- | -- | 100% | -- |
| PPV | -- | -- | 12% | -- | -- | -- | -- | -- |
| NPV | -- | -- | 92% | -- | -- | -- | 99% | -- |
| Correctly Classified | -- | -- | 91% | -- | -- | -- | 99% | -- |
| AUC | -- | -- | 0.63 | -- | -- | -- | 0.70 | -- |

Legend: **GI**: Gastrointestinal. **ROS**: Review of systems. **SLE**: systemic lupus erythematosus.
